# N-type calcium channel antibody-mediated autoimmune encephalitis: An unlikely cause of a common presentation^☆^^☆☆^^★^

**DOI:** 10.1016/j.ebcr.2013.06.001

**Published:** 2013-06-28

**Authors:** Leslie Finkel, Sookyong Koh

**Affiliations:** a Neurology and Epilepsy, Ann and Robert H. Lurie Children's Hospital of Chicago, 225 E. Chicago Avenue, Chicago, IL 60611, USA; b Neurobiology Program, Ann and Robert H. Lurie Children's Hospital of Chicago Research Center, Department of Pediatrics, Northwestern University, Feinberg School of Medicine, Chicago, IL 60614, USA

**Keywords:** IVIG, intravenous immunoglobulin, CSF, cerebrospinal fluid, EEG, electroencephalogram, CNS, central nervous system, MRI, magnetic resonance imaging, CT, computed tomography, SPECT, single photon emission computed tomography, NMDAR, N-methyl d-aspartate receptor, Encephalitis in children, Limbic encephalitis, Inflammatory epilepsy

## Abstract

We report, to our knowledge, the only known pediatric case with encephalopathy and significantly elevated titers of N-type voltage-gated calcium channel antibody (N-type VGCC). The patient, an 8th grader, was previously healthy and presented with a one-week history of confusion, aphasia, transient fever, headaches, and dizziness. An underlying autoimmune process was suspected because of inflammatory changes in the brain MRI and multiple focal electrographic seizures captured in the EEG in the absence of CSF pleocytosis. Within 24 h of presentation, the patient was empirically started on immune-modulatory therapy, and a full recovery was achieved within 3 months of the initial presentation. Immune therapy included high-dose intravenous (IV) methylprednisolone followed by a 2-week course of dexamethasone and 2 monthly courses of IV immunoglobulin (IVIG). He was also treated with anticonvulsants for one month. No tumor has been found to date. There is a paucity of reports on autoimmune epilepsy or encephalopathy associated with N-type VGCC. Complete resolution of brain lesion, seizure freedom, and full recovery of function following early and aggressive immunotherapy demonstrate that a high index of suspicion is crucial for early recognition and treatment of autoimmune encephalitis.

## 1 Introduction

The etiology of encephalitis often remains a mystery. Fortunately, we are now able to test for an increasing number of antibodies that offer a definitive diagnosis for cases that would have been previously categorized as viral or idiopathic encephalitis [1]. The California Encephalitis Project found that the frequency of autoimmune encephalitis was greater than any single viral etiology [2]. Anti-N-methyl d-aspartate receptor (NMDAR) encephalitis was, in fact, the most frequent cause of immune mediated encephalitis in the pediatric population [2]. Any child who presents with memory deficits, aphasia, seizures, movement disorders, or behavioral changes of a subacute nature should have autoimmune encephalitis in the differential diagnosis. The initial presentation of these patients can vary depending on the receptor being targeted, but it is crucial to always consider immune-mediated encephalitis because of the potential for a successful outcome with prompt treatment.

The case we present is of a young man with an elevated titer of N-type voltage-gated calcium channel antibody and a relatively acute change in mental status including memory deficits, mood changes, and transient fever. Our initial instinct was to look for a bacterial or viral etiology. When the lumbar puncture was benign, however, an autoimmune etiology became the leading consideration in our differential diagnosis. Entities such as fever-induced refractory epileptic encephalopathy in school-aged children (FIRES) or Rasmussen encephalitis were also considered. Fever-induced refractory epileptic encephalopathy in school-aged children is triggered by fever and an underlying inflammatory response in the suspected culprit [3]. Although our patient's clinical history was preceded by fever, his clinical course and prompt positive response to steroids and IVIG are different from patient with FIRES [3], [4] because those patients present with drug-resistant status epilepticus, CSF pleocytosis, and perisylvian/mesial temporal lobe abnormalities [4]. Rasmussen encephalitis is a presumed inflammatory encephalopathy and a recognized cause of an intractable focal epilepsy or epilepsia partialis continua (EPC). In most cases, only one hemisphere is involved, and patients progress to have weakness on the contralateral side. Our young man's initial MRI scan was reminiscent of this type of unilateral hemispheric dysfunction, although his lack of EPC or clinically evident focal seizures or weakness led us away from this diagnosis. The etiology of Rasmussen encephalitis remains largely unknown, although it is considered a prototype of immune-inflammatory epilepsy syndrome, and multiple mechanisms have been proposed [2]. Patient's with Rasmussen encephalitis also do not typically respond readily to immunotherapies, often requiring hemispherectomy to afford seizure freedom [5].

Treatment for encephalitis often needs to be initiated before the results of confirmatory tests are available. Typically, intravenous immunoglobulin and high-dose steroids are used in tandem as initial therapy. Supportive care and treatment of seizures also play a crucial role in a favorable outcome. Finding an underlying malignancy in an adult is more likely than in the pediatric population, but children should still be screened because a dramatic response can be seen after tumor removal. There are many types of autoimmune encephalitis, and outcomes vary. Our case demonstrates that an autoimmune etiology must always be considered in patients who present with acute behavioral change because of the potential for improved outcome with early administration of immunotherapy [5].

## 2 Patient presentation

A previously healthy and normally developing 14-year-old boy presented with one week history of intermittent confusion, memory deficit, emotional liability, inactivity, and transient fever and complained of headaches, dizziness, and inability to talk. His brother had a febrile illness earlier in the week, and the patient experienced one day of fever, with a maximum temperature of 101.7 °F a day before the acute change in his mental state. Examination on admission was remarkable for an ill-appearing, tearful boy with significant deficits in memory, attention, language, and orientation. He was oriented to person and place but not time and had difficulty naming simple objects (“bone” instead of “ball”). His digit span was only 2 forward, and he could not follow the directions for backward digit span.

**Fig. 1:**
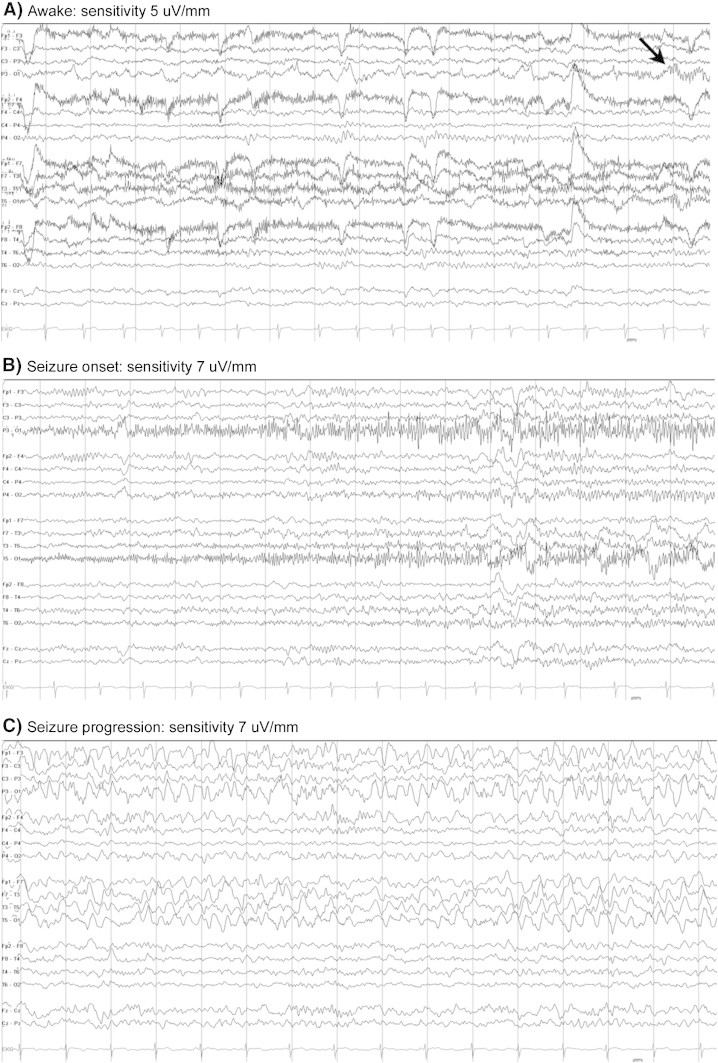
Interictal and ictal activities during overnight vEEG monitoring demonstrated marked left posterior dysfunction and epileptogenicity. A.Intermittent, prominent slowing noted in the left hemisphere maximum in the temporal and occipital regions. A moderate fast rhythm, 18–20 Hz, was often noted in the left occipital region (arrow). Note a lack of posterior dominant rhythm (PDR) on the left and preserved 9-Hz PDR on the right hemisphere.B.Multiple brief electrographic seizures were captured from the awake and sleep states. The seizure onset was subtle and stuttering. Ictal EEG correlate suggests posterior (left temporal/occipital) seizure origin. Posterior maximum left hemisphere fast activity spread throughout the left hemisphere and evolved in amplitude and frequency.C.Evolution of rhythmic delta activity spreading throughout the left hemisphere.

**Fig. 2:**
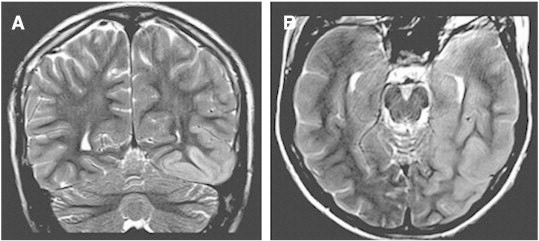
MRI of brain. There is cortical thickening with mildly associated increased T2 signal within the left posterior temporal and mesotemporal lobe with involvement of the hippocampal and parahippocampal gyri. A. Coronal T2-weighted image; B. axial T2-weighted image.

Head computed tomography (CT) was unremarkable, and a lumbar puncture (LP) was performed to rule out meningoencephalitis, which showed only 5 white blood cells (WBCs) with normal glucose and protein (Table 1). Overnight video electroencephalogram (vEEG) showed marked left hemisphere dysfunction, a lack of a posterior dominant rhythm on the left and epileptogenicity with temporal and occipital maximum. Electroencephalogram captured multiple, 20- to 30-second-long, subtle/stuttering electrographic seizures emanating from the left hemisphere (Fig. 1). Magnetic resonance imaging (MRI) revealed cortical thickening involving the left hippocampal and parahippocampal gyri and temporal lobes with associated abnormal FLAIR signal and restriction. The imaging pattern was most suggestive of an underlying inflammatory process such as encephalitis or prolonged seizure activity (Fig. 2).

**Table 1:** Laboratory data.

Lab test	Result
CBC	WBC: 5.58 thou/μL; Hgb: 14.5g/dL; Hct: 43%; Plt: 203 thou/μL
CRP, ESR	< 0.5 mg/dL (0–0.8 mg/dL), 8 mm/h (0–20 mm/h)
Urine drug panel	Unremarkable
CSF from lumbar puncture #1	Protein: 28 mg/dL (20–80 mg/dL); glucose: 68 mg/dL (37–65 mg/dL); RBC: < 1/mm^3^ (0–10/mm^3^); WBC: 5/mm^3^ (0–5/mm^3^), 1% neutrophils (0%), 87% lymphocytes (63–99%)
CSF from lumbar puncture #2	Protein: 32 mg/dL; glucose: 74 mg/dL; RBC: < 1/mm^3^; WBC: 1/mm^3^, 1% neutrophils, 92% lymphocytes
HSV PCR, meningoencephalitis panel, EBV, HHV-6	Negative
GAD 65 antibody	0.00 nmol/L (< 0.02 nmol/L)
Antithyroid antibodies	Negative
Paraneoplastic antibody evaluation (serum)	Anti-N-type voltage-gated calcium channel antibody = 0.26 nmol/L (≤ 0.03 nmol/L)
NMDA receptor antibody serum	Negative

CBC, complete blood count; WBC, white blood cell; Hgb, hemoglobin; Hct, hematocrit; Plt, platelet; RBC, red blood cell; CRP, C-reactive protein; ESR, erythrocyte sedimentation rate; CSF, cerebrospinal fluid; HSV, herpes simplex virus; PCR, polymerase chain reaction; EBV, Epstein–Barr virus; HHV, human herpes virus; GAD, glutamate decarboxylase; NMDA, N-methyl d-aspartic acid.

### 2.1 Treatment and hospital course

**Fig. 3:**
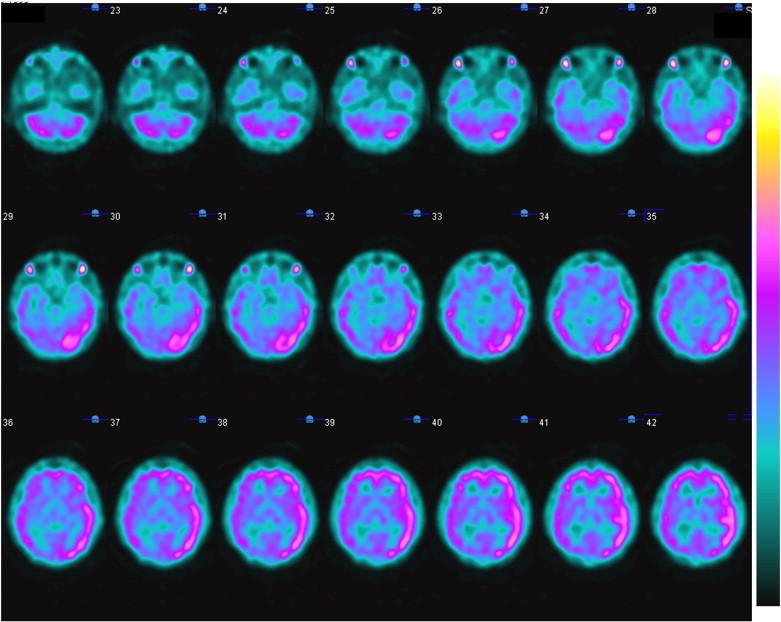
Perfusion SPECT imaging of the brain performed with Tc 99m EDC (Neurolite). 2D grayscale and color maps were produced using iterative reconstruction with and without attenuation correction. There is asymmetrically increased radiotracer uptake throughout the left cerebral hemisphere in comparison to the right, which is most pronounced in the left occipital and posterior parietal lobes. These findings correspond to the areas of signal abnormality and gyral swelling in the left cerebral hemisphere on MRI and suggest that images were acquired during seizure activity, likely an ictal SPECT.

One gram of intravenous (IV) methylprednisolone (Solumedrol) was started soon after MRI and EEG and within 24 h of admission. The patient was also loaded with fos-phenytoin and started on maintenance fos-phenytoin and topiramate. Intravenous Solumedrol was continued for a total of 3 days followed by dexamethasone (6 mg TID × 5 days, 6 mg BID × 5 days, 6 mg daily × 5 days, 3 mg daily × 5 days). He continued to demonstrate waxing and waning mental status after a high-dose Solumedrol burst. Repeat video-EEG monitoring on day 9 no longer detected seizures but continued to show absence of a posterior dominant rhythm on the left. Single photon emission computed tomography scan was performed on day 9 to address continued concern for ongoing subclinical seizure activity that was not being captured on EEG. It demonstrated an asymmetric hyperperfusion throughout the left cerebral hemisphere with posterior predominance, corresponding to the findings seen on MRI, which may have reflected ongoing seizure activity (Fig. 3). Repeated doses of lorazepam were given at times of aphasia, confusion, and sleepiness and produced a transient clinical improvement. The patient was, therefore, started on a daily dose of clobazam. Repeat MRI of the brain (with and without contrast) on day 10 of the hospital stay displayed marked improvement; only subtle T2 hyperintensity, with minimal cortical thickening in the left occipital and inferior temporal lobes, was noted. The repeat lumbar puncture on day 10 was again unremarkable (Table 1). The patient was given 2 doses of 1-g/kg intravenous immunoglobulin (IVIG) on days 11 and 12 of hospitalization. On day 18, he was transferred to inpatient rehabilitation with a plan for clobazam monotherapy (10 mg BID).

One month after initial presentation, the paraneoplastic panel came back positive for N-type calcium channel binding antibody (0.26 nmol/L [≤ 0.03 nmol/L]). One additional dose of 1-g/kg IVIG was given. Testicular ultrasound showed 1-cm hypoechogenicity in the upper pole of the right testis and bilateral microlithiasis. Repeat ultrasound and urology follow-up produced no evidence for malignancy. He had completely normal neurological exam at his 3-month follow-up visit. The most recent MRI of the brain, completed four months after the initial MRI, demonstrated complete/near-complete resolution of abnormal T2 hyperintense signal and cortical thickening. Our patient has been able to go back to school and has not had any notable residual deficits.

## 3 Discussion

Early recognition of encephalopathy being related to an underlying autoimmune mechanism in our patient led to expedited immune-modulatory therapy and subsequent favorable outcome. Detection of elevated antibody titer of N-type VGCC in his serum and his positive response to steroids and IVIG point towards an autoimmunity as the cause of his symptoms [6]. A case report of two boys with encephalitis of unknown etiology demonstrated the positive effect of immunotherapy even in the absence of a known antibody as the cause [7]. Patients with immune-mediated encephalitis can present with the wide spectrum of symptoms including psychosis, catatonia, alterations of behavior and memory, seizures, abnormal movements, and autonomic dysregulation [2]. A high degree of suspicion for an autoimmune etiology may, therefore, lead to an early administration of immune-modulatory therapy and a better outcome [5]. In cases of underlying malignancy, it is important to also treat and remove the malignant tumor for successful outcome [5]. As noted in the literature on anti-NMDAR encephalitis, over 75% of patients have significant recovery of neurologic issues when treated early with immunotherapy and tumor removal whenever applicable [8].

At the time of initial presentation, differential diagnosis included a parainfectious or infectious etiology in our patient. The two lumbar punctures, however, were unrevealing (Table 1). Also, we have no reason, at this point, to suspect an underlying malignancy. Close follow-up is necessary as encephalitis often develops prior to the diagnosis of cancer. The adult guidelines recommend repeating the screening in 3–6 months if primary screening is negative and then every 6 months for 4 years, except in Lambert–Eaton myasthenia syndrome (LEMS). For LEMS, follow-up for 2 years is recommended [9].

We are unaware of another case report of a child with encephalitis secondary to N-type calcium channel receptor antibody. A case report was found of a 65-year-old woman with small cell lung cancer who presented with seizure, confusion, dizziness, and lethargy and was subsequently found to have anti-N-type voltage-gated calcium channel titer elevation (0.42 nmol/L [< 0.03 nmol/L]) [10]. N-type voltage-gated calcium channel antibody does not have a well-described association with a specific paraneoplastic syndrome or underlying malignancy [10], whereas in other antibodies associated with characteristic CNS syndromes, the presence of various tumors has been found as the underlying source of the antibody production [6]. N-methyl d-aspartate receptor encephalitis, for example, is often found in association with ovarian teratoma [11]. Lambert–Easton myasthenic syndrome (LEMS) and paraneoplastic cerebellar degeneration are commonly associated with small cell lung cancer (SCLC) [6], [12]. Lambert–Easton myasthenic syndrome is associated with voltage-gated calcium channels (most of the P/Q type with N-type found in about 35%) [12]. The P/Q-type VGCC is also associated with paraneoplastic cerebellar degeneration. Other synaptic proteins targeted in limbic encephalitis or autoimmune epilepsies include α-amino-3-hydroxy-5-methyl-4-isoxazolepropionic acid receptor (AMPAR), γ-aminobutyric acid (GABA) receptor, voltage-gated potassium channel complex (VGKC), glutamic acid decarboxylase (GAD) 65, collapsin response-mediated protein 5 (CRMP5), and ganglionic acetylcholine receptor [1], [12]. Limbic encephalitis is more often a paraneoplastic syndrome in adults, while children are far less likely to have an underlying malignancy [12].

In summary, we present this unique case of a young man with encephalitis and high antibody titers of N-type voltage-gated calcium channel. Our case underscores the importance of early immune-modulatory therapy when an underlying autoimmune process is suspected. Patients presenting with new onset memory deficits, behavioral changes, and seizures of unknown etiology should have autoimmune encephalitis at the top of their differential diagnosis. Immunotherapy can be started prior to confirmation, and the timely treatment of these patients can facilitate improved outcome.

## Conflict of interest

None.
